# Ictal–interictal continuum and status epilepticus: Two sides of the same coin? A prospective magnetic resonance imaging study

**DOI:** 10.1002/epi.70131

**Published:** 2026-02-02

**Authors:** Pilar Bosque‐Varela, Lukas Machegger, Wanda Lauth, Panagiota Eleni Tsalouchidou, Susanne Knake, Georg Zimmermann, Nicolas Jannone‐Pedro, Giada Giovannini, Stefano Meletti, Adrian Ridski Harsono, Fabio Rossini, Markus Leitinger, Johannes Pfaff, Sándor Beniczky, Eugen Trinka, Giorgi Kuchukhidze

**Affiliations:** ^1^ Department of Neurology, Neurocritical Care, and Neurorehabilitation Christian Doppler University Hospital, member of European Reference Network EpiCARE, and Center for Cognitive Neuroscience, Paracelsus Medical University Salzburg Austria; ^2^ Neuroscience Institute, Christian Doppler University Hospital, Center for Cognitive Neuroscience Salzburg Austria; ^3^ Department of Neuroradiology Christian Doppler University Hospital, Paracelsus Medical University Salzburg Austria; ^4^ Department of Mathematics Paris‐Lodron University Salzburg Austria; ^5^ Epilepsy Center Hessen, Department of Neurology Philipps University Marburg Germany; ^6^ Second Department of Neurology Attikon University Hospital, National and Kapodistrian University Athens Greece; ^7^ Team Biostatistics and Big Medical Data, IDA Lab Salzburg, Paracelsus Medical University Salzburg Austria; ^8^ Research and Innovation Management, Paracelsus Medical University Salzburg Austria; ^9^ Department of Clinical Neurophysiology University and Polytechnic La Fe Hospital, member of European Reference Network EpiCARE Valencia Spain; ^10^ Unit of Clinical Neurophysiology OCB Hospital Modena Italy; ^11^ Neurophysiology and Epilepsy Division, Department of Neurology, Faculty of Medicine University of Indonesia Jakarta Indonesia; ^12^ Department of Clinical Medicine University of Copenhagen Copenhagen Denmark; ^13^ Department of Clinical Neurophysiology Copenhagen University Hospital Rigshospitalet Copenhagen Denmark; ^14^ Danish Epilepsy Center Dianalund Denmark; ^15^ Karl Landsteiner Institute for Clinical Neurosciences Salzburg Austria

**Keywords:** diffusion‐restricted lesion, electroencephalography, lateralized periodic discharges, peri‐ictal MRI abnormalities

## Abstract

**Objective:**

Status epilepticus (SE) is the most severe expression of seizures, encompassing both SE with prominent motor symptoms and nonconvulsive SE (NCSE). Ictal–interictal continuum (IIC), an electroencephalographic phenomenon, is characterized by periodic discharges (PD), spike‐and‐waves or sharp‐and‐waves (SW), or lateralized rhythmic delta activity (LRDA). Peri‐ictal magnetic resonance imaging (MRI) abnormalities (PMA) may offer a potential surrogate marker for ictal activity, yet their association with IIC remains unclear. We aimed to investigate the occurrence of PMA in patients with SE and IIC, and to determine the relationship between IIC patterns and PMA through a latent cluster analysis (LCA).

**Methods:**

In a prospective cohort study, 223 adult patients diagnosed with SE or IIC underwent electroencephalography (EEG) and MRI within 48 h of diagnosis. Patients were stratified into two groups: the IIC group and SE group. PMA were assessed using the following MRI sequences: diffusion‐weighted imaging, fluid‐attenuated inversion recovery, and arterial spin labeling. LCA was performed to identify classes based on etiology, EEG patterns, and their localization.

**Results:**

PMA were as frequent in patients of the IIC group (23/49, 47%) as in patients of the SE group (64/149, 43%, *p* = .37). In the IIC group, peri‐ictal hyperperfusion was more frequently associated with lower frequency PD/SW (.5–1 Hz; 12/19, 63%), followed by LRDA (4/13, 31%) and higher frequency PD/SW (>1–2.5 Hz; 4/17, 24%). LCA revealed two classes; Class 1, characterized by nonunilateral high‐frequency PD/SW and triggering factors in epilepsy, had fewer PMA (18%) as compared to Class 2, characterized by predominantly unilateral low‐frequency PD/SW and diverse etiologies (50%; odds ratio = 5.79, *p* = .02).

**Significance:**

PMA occurrence in IIC aligned closely with that in SE, suggesting an overlap between IIC and SE and raising the critical question of whether patients with IIC may have NCSE. We propose an etiology‐driven approach for EEG interpretation in IIC, which may enhance diagnostic accuracy and treatment strategies.

**Key points**

- Patients with IIC and SE have similar rates of PMA, suggesting that IIC could actually be nonconvulsive SE.
- Low‐frequency PD/SW are more commonly associated with peri‐ictal hyperperfusion, reflecting possible increased metabolic demand.
- Latent cluster analysis based on EEG and etiology may help in more accurate interpretation of IIC.

## 1 INTRODUCTION

Status epilepticus (SE) stands as the most severe manifestation of seizures, encompassing SE with prominent motor symptoms (including bilateral tonic–clonic SE, i.e., convulsive SE) and nonconvulsive SE (NCSE).^1^ “SE is a condition which can have long‐term consequences (after time point t2), including neuronal death, neuronal injury, and alteration of neuronal networks, depending on the type and duration of seizures.”^1^ The complexity of the electroencephalogram (EEG) correlates of NCSE often poses diagnostic challenges. When diagnostic EEG criteria are not fulfilled, terms such as “possible (electrographic) NCSE” (according to the Salzburg Consensus Criteria^2^) or “ictal–interictal continuum (IIC),” as in the updated American Clinical Neurophysiology Society (ACNS) Standardized Critical Care EEG Terminology 2021,^3^ are introduced.

ACNS defined three EEG patterns as IIC: (1) any periodic discharges (PD) or spike‐and‐waves or sharp‐and‐waves (SW) at a frequency of >1.0–2.5 Hz; (2) PD or SW at a frequency of .5–1.0 Hz with a plus modifier or fluctuation; and (3) lateralized rhythmic delta activity (LRDA) averaging >1 Hz with a plus modifier or fluctuation. Thus, IIC is a purely electrographic phenomenon, representing the borderland or a “boundary syndrome” of SE,^1^, ^4^, ^5^ with no clear distinction between ictal and interictal EEG activity, hence the term IIC. In clinical practice, however, clear differentiation between “ictal” and “interictal” may be crucial for taking therapeutic decisions.^6^, ^7^, ^8^ On the one hand, failure to recognize EEG patterns that are more ictal, potentially indicative of NCSE, can lead to longer lasting episodes, potentially exceeding the time point t2 and therefore increasing the risk of long‐term consequences.^1^, ^9^, ^10^ On the other hand, patients with more benign EEG patterns might benefit from less aggressive interventions and therefore a lower burden of treatment.^8^

Peri‐ictal magnetic resonance imaging (MRI) abnormalities (PMA) are imaging correlates of SE, occurring in up to 45% of patients.^11^, ^12^, ^13^ PMA may play a role in diagnosing NCSE in cases of equivocal EEG patterns.^14^ Thalamocortical peri‐ictal MRI hyperperfusion, for instance, is considered a potential diagnostic biomarker of NCSE.^15^ PMA are seen also in patients with IIC, as demonstrated in small retrospective cohorts.^16^ They are associated with PD and rhythmic delta activity of different frequencies.^15^ In critically ill patients, LRDA of frequency higher than 1.5 Hz is linked to occurrence of seizures.^17^ Frequencies greater than 2 Hz for periodic and lateralized rhythmic patterns, among other parameters, were associated with the increased risk of seizures in a widely utilized 2HELPS2B score.^18^ PMA may also serve as a prognostic biomarker of SE; their occurrence may be associated with the unfavorable clinical outcome^19^ and may predict potential cerebral injury.^10^, ^20^ There is a significant gap in identifying reliable biomarkers of a possible acute brain injury in patients with IIC.

The main objective of this study was to compare the occurrence of PMA in patients with IIC and SE. Furthermore, we aimed to determine which IIC patterns were most frequently associated with PMA, a potential surrogate marker for SE‐related neuronal injury. We hypothesized that different EEG patterns in IIC may have distinct associations with PMA.

## 2 MATERIALS AND METHODS

### 2.1 Study design

This prospective, single‐center cohort study included adult patients (≥18 years old) with SE or IIC recruited at the Department of Neurology and the Neurointensive Care Unit of the Department of Neurosurgery, Christian Doppler University Hospital, Paracelsus Medical University, Salzburg, Austria.

### 2.2 Participants

Inclusion criteria were as follows: adult patients with SE or IIC who underwent MRI with a standard protocol (Appendix S1) within the first 48 h after the diagnosis.

Exclusion criteria were as follows: patients with global cerebral hypoxia due to cardiac arrest, patients with acute central nervous system (CNS) lesions overlapping with or indistinguishable from PMA (e.g., mitochondrial encephalomyopathy, lactic acidosis, and strokelike episodes [MELAS]), and patients with low‐quality MRI affected by movement artifacts.

In all patients, MRI was performed on a 3‐T machine (Achieva dStream, Philips Medical Systems) at the Institute of Neuroradiology of our institution.

Patients were stratified into two groups.

### 2.3 IIC group

Patients were diagnosed with IIC based on ACNS criteria.^3^ Subcategories included the following:

- Patients with PD or SW in the frequency range >1.0–2.5 Hz.
- Those with PD or SW in the frequency range .5–1.0 Hz, associated with either a modifier or fluctuation.
- Patients exhibiting LRDA of >1 Hz along with a modifier or fluctuation.

### 2.4 SE group

Patients were diagnosed with SE based on the criteria of the International League Against Epilepsy^1^ and the Salzburg EEG diagnostic criteria for NCSE implemented into the ACNS criteria.^2^, ^3^

Patients transitioning from SE with prominent motor symptoms to NCSE, or to IIC, underwent a separate analysis to ensure that PMA occurrence in the primary analysis (IIC vs. SE) could not be attributed to the preceding prominent motor symptoms.

### 2.5 Electroencephalography

Ictal EEG recordings based on the international 10–20 system were available in all patients, except for those with convulsive SE (*n* = 90) in whom EEG was not performed for diagnostic purposes. The duration of the EEG recordings was at least 20 min for all patients. The analysis of EEG patterns was based on the validated 2013 Salzburg criteria^21^ and the 2021 ACNS terminology.^3^ Patients were categorized based on key features such as PD, SW, LRDA, presence of modifiers and fluctuation, and response to an antiseizure medication (ASM) trial.^22^ Patients who exhibited these EEG patterns along with both clinical and EEG response to an ASM trial were classified as NCSE, whereas those who did not respond to the ASM trial were categorized as IIC. Localization of EEG activity was regarded as unilateral or nonunilateral (including generalized, bilateral and multifocal patterns). In the case of the IIC group, four external reviewers blinded to clinical data (G.G., P.E.T., N.J.‐P., A.R.H.) assessed the EEGs.

### 2.6 MRI data

All patients underwent MRI within the first 48 h after the diagnosis of SE or IIC with a standard protocol dedicated to SE (Appendix S1), as this is the optimal time window for capturing PMA.^11^, ^23^

The presence of PMA was assessed in the following MRI sequences: diffusion‐weighted imaging (DWI), fluid‐attenuated inversion recovery (FLAIR), and arterial spin labeling (ASL).

Diffusion‐restricted lesions and hyperintense signal in FLAIR were attributed to PMA if they fulfilled at least one of the following criteria: (1) PMA affecting brain areas such as pulvinar of thalamus or hippocampus in combination with cortex, not respecting vascular territories^12^; (2) in the case of diffusion‐restricted lesions, they were classified as PMA if the quantification analysis revealed a signal intensity ratio lower than 1.495 for DWI and higher than .735 for apparent diffusion coefficient, based on a previous study by Machegger et al.^13^; and (3) presence of a simultaneous hyperperfusion in ASL. MRI abnormalities were not classified as PMA if they were primary CNS lesions such as cerebral infarcts, tumors, or encephalitis.

MRI assessment was conducted by two independent reviewers (L.M., G.K.) blinded to clinical data in the SE group and in the spectrum of SE with prominent motor symptoms to NCSE or to IIC. For the IIC group, two external independent reviewers were involved (S.M., P.E.T.). Disagreements were resolved by a third reviewer (J.P.).

### 2.7 Statistical analysis

In the initial stage of the analysis, medians, interquartile ranges (IQRs), and absolute and relative frequencies were calculated to characterize the overall study population and to observe differences between two study groups: IIC and SE. To investigate those differences further, the Kruskal–Wallis test was employed in metric and ordinal variables between more than two groups. In the event of a significant result, the Dunn test was performed as a post hoc test. If only two subgroups were to be compared, the Mann–Whitney *U*‐test was applied. The Fisher exact test was utilized to detect differences in nominal and binary variables. A global approach was selected for the comparison of more than two groups (global Fisher exact test), with pairwise Fisher exact tests serving as post hoc tests in the event of a significant result. To gain further insight into the discrepancies in frequencies, the odds ratios (ORs) and their 95% confidence intervals (CIs) were also calculated. In the event of post hoc tests, the Bonferroni–Holm correction was utilized to adjust for multiplicity. The two‐sided significance level *α* = .05 was used for all hypothesis tests. In a further part, a latent cluster analysis (LCA)^24^ was performed to find subgroups, with the premise of recognizing patterns and features related to the occurrence of PMA without being bound by the limitations of the group definitions mentioned above (IIC and SE). Therefore, the following variables were included: etiology, EEG location, and EEG pattern. Etiology was divided into three categories based on the proposed classification by Lattanzi et al.^25^: (1) acute‐triggering factors in epilepsy; (2) other acute etiologies, such as acute primary, secondary, or toxic–metabolic; and (3) a combined category of remote, progressive, or unknown etiologies. Etiology was divided into three categories based on the classification by Lattanzi et al.,^25^ separating acute‐triggering factors in epilepsy from acute symptomatic etiologies, as these etiologies are most likely to be associated with good and unfavorable outcomes, respectively. Remote, progressive, and unknown etiologies were grouped together due to limited sample sizes for the LCA, which did not allow a more granular subgroup analysis. The location of the investigated activity on EEG was classified as either unilateral or nonunilateral. EEG patterns were categorized into PD or SW of different frequency bands (.5–1 Hz, >1–2.5 Hz, >2.5 Hz), and LRDA. In this part of the analysis, only patients with NCSE (*n* = 59) and IIC (*n* = 49) were included, as ictal EEG was not available in patients with convulsive SE (*n* = 90). The optimal number of classes, ranging from two to four, was selected based on the model exhibiting the lowest Bayesian information criterion value. The model itself was computed 500 times with different starting points to find the parameter estimates corresponding to the model with the greatest log‐likelihood. The resulting classes were then compared using abovementioned methods. To assess stability of the latent class solution, a subsampling analysis was conducted. The final model (K = 2 classes) was re‐estimated in 200 random subsamples comprising 80% of the original data. Agreement with the full‐sample solution was quantified using the adjusted Rand index (ARI).^26^ All calculations were carried out using the statistical software R (Version 4.3.2).

### 2.8 Standard protocol approvals, registrations, and patient consents

The Ethics Committee of the Region of Salzburg approved this study on human subjects (approval number 415‐E/2422). All patients gave informed consent for MRI, which was performed in the framework of diagnostic workup and a current study.

This study was conducted based on the STROBE (Strengthening the Reporting of Observational Studies in Epidemiology) reporting guidelines.

## 3 RESULTS

**FIGURE 1:**
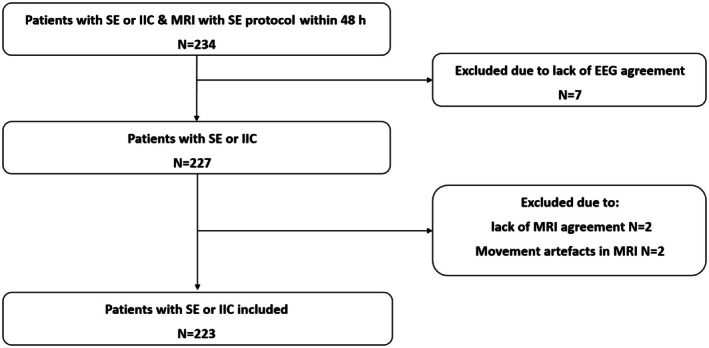
Flowchart of patients' selection process. Between February 2019 and March 2024, 710 patients with clinically suspected status epilepticus (SE) or an electroencephalographic (EEG) report of ictal–interictal continuum (IIC) were prospectively enrolled. Of these, 420 (59%) underwent magnetic resonance imaging (MRI) during the acute phase. Patients with cerebral hypoxia due to cardiac arrest (*n* = 9) and mitochondrial encephalomyopathy, lactic acidosis, and strokelike episodes (*n* = 3), as well as those with MRI performed after 48 h (*n* = 97) or nonprotocol SE MRI (*n* = 77) were excluded. MRI was not performed in 290 of 710 (41%). Disagreements between the reviewers of EEG led to exclusion of seven patients and between the reviewers of MRI to four exclusions. Eventually, 223 patients were included in the final analysis.

Between February 2019 and March 2024, 710 patients with clinically suspected SE or IIC in an EEG report were prospectively enrolled. Of these patients, 420 (59%) underwent MRI during the acute phase. For further analysis, 234 patients with either SE or IIC who had MRI within the first 48 h of diagnosis were selected after excluding patients with cerebral hypoxia due to cardiac arrest (*n* = 9) and MELAS (*n* = 3), as well as those with MRI performed after 48 h (*n* = 97) or nonprotocol MRI (*n* = 77; Figure 1).

MRI was not performed in 290 of 710 (41%) patients for the following reasons: previous episodes of SE due to noncompliance (*n* = 98), terminal illness (*n* = 99), known causes of epilepsy (*n* = 82), implants incompatible with MRI (*n* = 10), and extreme scoliosis (*n* = 1).

### 3.1 Assessment of EEG and MRI

Evaluation of IIC patterns showed moderate agreement (kappa = .43, 95% CI = .21–.66), leading to the exclusion of seven patients due to discordance between the reviewers. MRI assessment demonstrated substantial agreement, with kappa value of .78 (95% CI = .63–.93) for the IIC group and .78 (95% CI = .68–.88) for the SE group, resulting in four exclusions due to motion artifacts or lack of consensus. Eventually, 223 patients were included in the final analysis (Figure 1). Demographic and clinical data of patients are detailed in Table 1.

**TABLE 1:** Demographic and clinical characteristics of the entire cohort (*N* = 223).

Variable	Value
Age, years, median (IQR)	68 (23)
Sex, female	112 (50%)
PMA spectrum	
PMA total	105 (47%)
Peri‐ictal hyperperfusion	93 (42%)
Diffusion restriction	66 (30%)
FLAIR hyperintensity	60 (27%)
SE/IIC	
IIC group	49 (22%)
SE group	149 (67%)
Spectrum of SE with prominent motor symptoms to NCSE or to IIC	25 (11%)
Etiology	
Acute‐triggering factors in epilepsy	23 (10%)
Acute primary CNS pathology	50 (22%)
Secondary CNS pathology	22 (10%)
Remote	57 (26%)
Progressive	52 (23%)
Electroclinical syndrome	1 (.44%)
Unknown	18 (8%)
Etiology categories	
Category A	23 (10.3%)
Category B	72 (32.3%)
Category C	128 (57.4%)
Time from onset to EEG, h, median (IQR)	5 (16)
Time from onset to MRI, h, median (IQR)	21 (32)
Time from EEG to MRI, h, median (IQR)	5 (24)

*Note*: The table shows the clinical, EEG, and MRI features of the entire cohort. Data are given as *n* (%) unless otherwise indicated. PMA include total PMA, peri‐ictal hyperperfusion, diffusion restriction, and FLAIR hyperintensity. SE and IIC groups are shown, including patients with SE with prominent motor symptoms. Etiologies are classified as follows: (A) acute‐triggering factors in epilepsy; (B) other acute etiologies, including primary, secondary, or toxic–metabolic causes; and (C) combined category of remote, progressive, or unknown etiologies.

Abbreviations: CNS, central nervous system; EEG, electroencephalography; FLAIR, fluid‐attenuated inversion recovery; IIC, ictal–interictal continuum; IQR, interquartile range; MRI, magnetic resonance imaging; NCSE, nonconvulsive SE; PMA, peri‐ictal MRI abnormalities; SE, status epilepticus.

Among these, 49 of 223 (22%) were categorized as IIC group and 149 of 223 (67%) as SE group. The spectrum of SE with prominent motor symptoms to NCSE or to IIC was observed in 25 of 223 (11%) patients.

The median time from the clinical onset to EEG did not differ between the IIC group (3.3 h, IQR = 15) and the SE group (5.5 h, IQR = 20.83; Mann–Whitney *U*‐test, *p* = .14). There was no significant difference in median time from EEG to MRI between groups (IIC group [*n* = 49]: 6.18 h, IQR = 23.5 vs. SE group [*n* = 59, as in 90 patients with convulsive SE no diagnostic EEG was done]: 20.4 h, IQR = 24.8; *p* = .277).

### 3.2 PMA occurrence: IIC group versus SE group

The rate of PMA and the spectrum of PMA abnormalities (peri‐ictal hyperperfusion, diffusion‐restricted lesions, and FLAIR hyperintensities) did not differ between groups. PMA were observed in 23 of 49 (47%) patients in the IIC group and in 64 of 149 (43%) patients in the SE group (OR = 1.17, 95% CI = .64–∞, *p* = .37; Table 2). A more detailed analysis showed that in the IIC group 20 of 49 (41%) had hyperperfusion, whereas 16 of 49 (33%) had diffusion restriction and 14 of 49 (29%) showed FLAIR hyperintensity. In the SE group, 56 of 149 (38%) had hyperperfusion, 37 of 149 (25%) had diffusion restriction, and 33 of 149 (22%) had FLAIR hyperintensity. These differences were not significant, as shown in Table 2.

**TABLE 2:** PMA spectrum in IIC versus SE.

PMA spectrum	IIC group, *n* = 49	SE group, *n* = 149	OR, 95% CI (*p*)
Peri‐ictal hyperperfusion	20 (41%)	56 (38%)	1.14, .62–∞ (.40)
Diffusion restriction	16 (33%)	37 (25%)	1.46, .75–∞ (.18)
FLAIR hyperintensity	14 (29%)	33 (22%)	1.40, .70–∞ (.23)

*Note*: The table shows the incidence of PMA subtypes (peri‐ictal hyperperfusion, diffusion restriction, and FLAIR hyperintensity) among patients with IIC and those with SE.

Abbreviations: CI, confidence interval; FLAIR, fluid‐attenuated inversion recovery; IIC, ictal–interictal continuum; OR, odds ratio; PMA, peri‐ictal MRI abnormalities; SE, status epilepticus.

In patients with SE with prominent motor symptoms, 35 of 90 (39%) had PMA. Of these, 30 of 90 (33%) showed hyperperfusion, 16 of 90 (18%) diffusion restriction, and 15 of 90 (17%) FLAIR hyperintensity. In the group of patients with NCSE, 18 of 42 (43%) had PMA, with 15 of 42 (36%) showing hyperperfusion, 11 of 42 (26%) diffusion‐restriction, and eight of 42 (19%) FLAIR hyperintensity.

### 3.3 PMA occurrence in patients with spectrum of SE with prominent motor symptom to NCSE versus spectrum of SE with prominent motor symptoms to IIC

PMA was observed in 11 of 15 (73%) patients with spectrum of SE with prominent motor symptoms to IIC and eight of 10 (80%) patients with spectrum of SE with prominent motor symptoms to NCSE, without significant differences (OR = .69, 95% CI = .05–6.38, *p* = 1.0). The rates of hyperperfusion, diffusion restriction, and FLAIR hyperintensity did not differ between these two groups, as shown in the Table S1.

### 3.4 PMA occurrence in relation to different EEG patterns in the IIC group

PMA rate was the highest in patients with PD or SW of low frequencies (.5–1 Hz) combined with modifiers or fluctuation, occurring in 13 of 19 (68%) patients. This was followed by LRDA, observed in five of 13 (38%) patients, and PD or SW in the high‐frequency range (>1–2.5 Hz), seen in five of 17 (29%) patients (*p* = .057).

Table 3 presents a comparison of PMA rates across different MRI sequences in relation to EEG patterns in the IIC group. Peri‐ictal hyperperfusion was most frequently observed in patients with low‐frequency PD/SW, documented in 12 of 19 (63%) patients, followed by LRDA (4/13, 31%) and PD/SW in the >1–2.5‐Hz range (4/17, 24%, *p* = .03). Pairwise comparisons of hyperperfusion rates in relation to three EEG patterns did not survive post hoc tests and did not differ significantly: (1) PD/SW .5–1 Hz versus >1–2.5 Hz (OR = 5.28, 95% CI = 1.07–31, *p* = .07), (2) PD/SW .5–1 Hz versus LRDA (OR = 3.69, 95% CI = .70–23.21, *p* = .30), and (3) PD/SW >1–2.5 Hz versus LRDA (OR = .70, 95% CI = .10–4.86, *p* = .70). Although no significant differences were found in occurrence of diffusion restriction or FLAIR hyperintensity among the three EEG patterns, there was a notable trend toward higher rates of PMA in patients with low‐frequency PD/SW (.5–1 Hz). Diffusion restriction was observed in 10 of 19 (53%) patients with low‐frequency PD/SW, compared to four of 17 (24%) in those with PD/SW of >1–2.5 Hz and two of 13 (15%) in those with LRDA (*p* = .06). FLAIR hyperintensity was found in eight of 19 (42%) of patients with PD/SW of .5–1 Hz, four of 17 (24%) with PD/SW of >1–2.5 Hz, and two of 13 (15%) with LRDA (*p* = .25).

**TABLE 3:** PMA spectrum in IIC group in relation to EEG patterns.

PMA spectrum	PD/SW >1–2.5 Hz, *n* = 17	PD/SW .5–1 Hz, *n* = 19	LRDA, *n* = 13	*p*
Peri‐ictal hyperperfusion	4 (24%)	12 (63%)	4 (31%)	.03^a^
Diffusion restriction	4 (24%)	10 (53%)	2 (15%)	.06
FLAIR hyperintensity	4 (24%)	8 (42%)	2 (15%)	.25

*Note*: This table shows the difference of incidences in types of PMA across the three different EEG patterns of IIC. Some patients with each EEG pattern had PMA in more than one MRI sequence (arterial spin labeling, diffusion‐weighted imaging, or FLAIR) or no PMA.

Abbreviations: EEG, electroencephalographic; FLAIR, fluid‐attenuated inversion recovery; IIC, ictal–interictal continuum; LRDA, lateralized rhythmic delta activity; MRI, magnetic resonance imaging; PD, periodic discharges; PMA, peri‐ictal MRI abnormalities; SW, spike‐and‐waves or sharp‐and‐waves.

^a^ Statistically significant.

### 3.5 Latent cluster analysis

The LCA identified two distinct classes, primarily based on EEG patterns, their localization, and their etiology. In this analysis, only IIC and NCSE patients were included (*n* = 133), as in patients with convulsive SE (*n* = 90) no ictal EEG was available. Class 1 (*n* = 11) was predominantly composed of patients with acute‐triggering factors in epilepsy as an etiology (Category A, 9/11, 81%); the remaining cases were associated with acute etiologies (Category B, 2/11, 19%).

**FIGURE 2:**
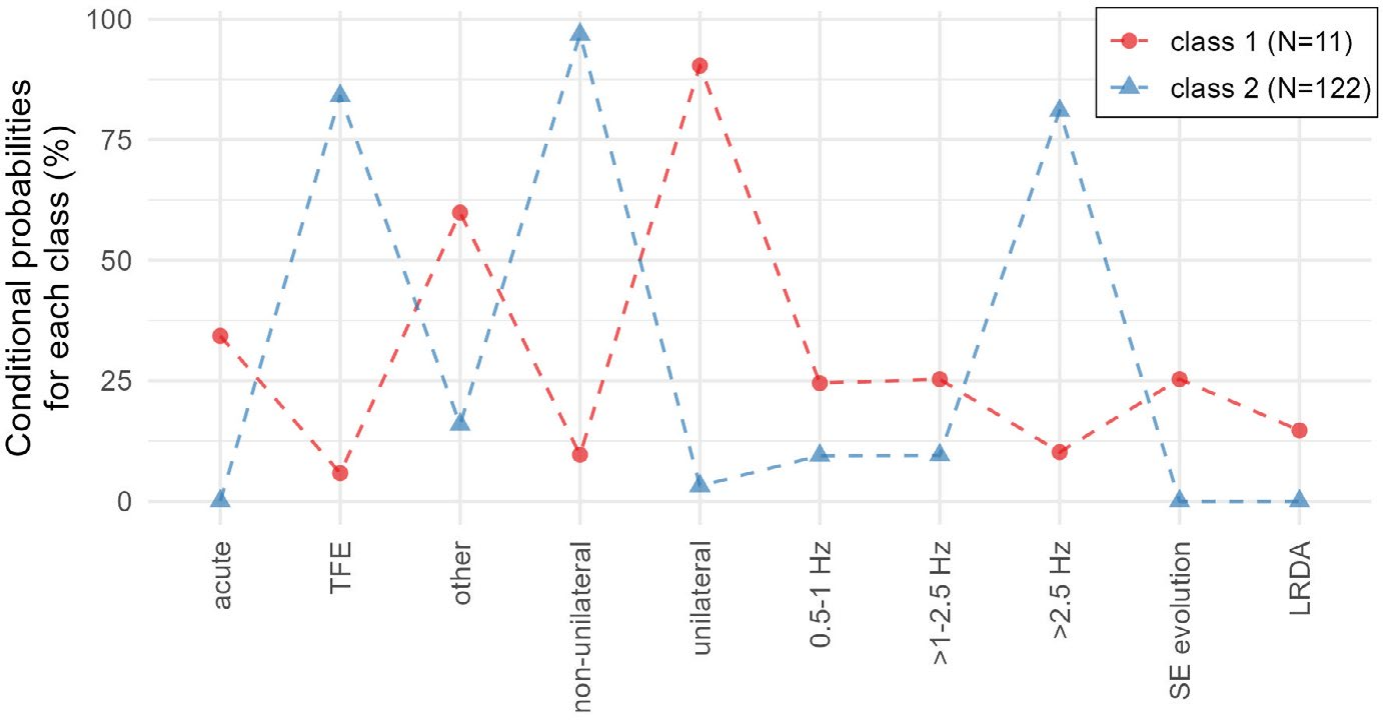
Latent cluster analysis of electroencephalographic (EEG) features. The latent cluster analysis identified two distinct classes based on EEG patterns, localization, and etiology. Class 1 is marked in red and was composed of 11 patients and characterized by high‐frequency periodic discharges (PD)/spike‐and‐waves or sharp‐and‐waves (SW) >2.5 Hz and predominantly acute‐triggering factors in epilepsy as etiology (81%). This class exhibited generalized EEG patterns in all patients. In contrast, Class 2, which is marked in blue, showed a higher number of patients (*n* = 122), had a broader distribution of etiologies, including acute, remote, progressive, or unknown causes, and was predominantly associated with low‐frequency PD/SW patterns (.5–1 Hz and >1–2.5 Hz). Class 2 exhibited predominantly unilateral EEG patterns (91%). LRDA, lateralized rhythmic delta activity; SE, status epilepticus; TFE, triggering factors in epilepsy.

Class 1 was dominated by high‐frequency PD/SW >2.5 Hz (9/11, 81%); low‐frequency patterns were rare: PD/SW at .5–1 Hz in one of 11 (9%) and at >1–2.5 Hz in one of 11 (9%) patients of each etiological category (Figure 2). Additionally, all EEG patterns in Class 1 were regarded as nonunilateral (*n* = 11).

Class 2 (*n* = 122) exhibited a broader distribution of etiologies, including seven of 122 (6%) patients with acute‐triggering factors in epilepsy (Category A), 42 of 122 (34%) with acute etiologies (Category B), and 73 of 122 (60%) with remote, progressive, or unknown etiologies (Category C). EEG patterns in Class 2 were predominantly of low‐frequency PD/SW (>1–2.5 Hz, 31/122, 25%; and .5–1 Hz, 30/122, 25%) or EEG with evolution (31/122, 25%). High‐frequency PD/SW of >2.5 Hz were observed in only 12 of 122 (10%) patients. LRDA was present in 18 of 122 (15%) patients. Notably, Class 2 exhibited predominantly unilateral EEG patterns (111/122, 91%); only 11 of 122 (9%) patients had a nonunilateral EEG pattern (Figure 2).

In summary, Class 1 was associated with high‐frequency and nonunilateral PD/SW patterns and predominantly acute‐triggering factors in epilepsy as an etiology. In Class 2, low‐frequency unilateral PD/SW predominated along with a mixture of different etiologies (Figure 2 and Table 4).

**TABLE 4:** Classes of latent cluster analysis and their features.

Variable	Class 1, *n* = 11	Class 2, *n* = 122
Etiology category		
A	9 (81%)	7 (6%)
B	2 (19%)	42 (34%)
C	0 (0%)	73 (60%)
EEG pattern location		
Nonunilateral	11 (100%)	11 (9%)
Unilateral	0 (0%)	111 (91%)
EEG pattern		
PD/SW >2.5 Hz	9 (81%)	12 (10%)
PD/SW .5–1 Hz	1 (9%)	30 (25%)
PD/SW >1–2.5 Hz	1 (9%)	31 (25%)
LRDA	0 (0%)	18 (15%)
Evolution	0 (0%)	31 (25%)

*Note*: This table displays the features of the different classes estimated by latent cluster analysis. Localization of EEG activity was regarded nonunilateral when generalized, bilateral, and multifocal patterns occurred. Etiology categories: (A) acute‐triggering factors in epilepsy; (B) other acute etiologies, such as acute primary, secondary, or toxic–metabolic; and (C) a combined category of remote, progressive, or unknown etiologies.

Abbreviations: EEG, electroencephalographic; LRDA, lateralized rhythmic delta activity; PD, periodic discharges; SW, spike‐and‐waves or sharp‐and‐waves.

PMA occurred in Class 1 in two of 11 (18%) patients and in Class 2 in 61 of 122 (50%) patients. This difference was statistically significant (OR = 5.79, 95% CI = 1.13–57.19, *p* = .02), indicating a higher likelihood of PMA occurrence in Class 2. Considering each MRI sequence, PMA also trended higher in Class 2, with peri‐ictal hyperperfusion (OR = 4.46, 95% CI = .87–44.05, *p* = .05) and diffusion restriction (OR = 6.64, 95% CI = .89–296.74, *p* = .05), although these results were not statistically significant. The OR for FLAIR hyperintensity was 5.59 (95% CI = .75–250.00, *p* = .10), suggesting a trend without significance (Table S2). The median ARI for the subsampling analysis was .93 (IQR = .44).

## 4 DISCUSSION

In this study, we found similar rates of PMA in patients with SE and IIC, indicating their overlap and leading to the following questions: (1) Which IIC patterns could be potentially considered to be NCSE? (2) Are IIC and EEG patterns in SE different entities or essentially a spectrum of one entity? (3) Is the 2.5‐Hz boundary applicable to all types of NCSE?

### 4.1 Which IIC patterns could be potentially considered to be NCSE?

In patients with IIC, low‐frequency PD/SW (.5–1 Hz) were more frequently associated with peri‐ictal hyperperfusion as compared to high‐frequency PD/SW (>1–2.5 Hz) or LRDA. There was a trend of more frequent occurrence of diffusion restriction and FLAIR hyperintensity in IIC patients with low‐frequency PD/SW (.5–1 Hz) as opposed to high‐frequency PD/SW (>1–2.5 Hz) and LRDA, although this was not statistically significant. In an LCA, the class with mainly acute, remote, progressive, or unknown etiologies and lateralized PD/SW of low frequencies (.5–1 Hz) was more commonly associated with PMA as compared to the class with acute‐triggering factors in epilepsy as a primary etiology and nonunilateral high‐frequency PD/SW (>1–2.5 Hz). The findings of this explorative analysis may suggest that these IIC patterns, previously considered to be a boundary syndrome of SE, particularly low‐frequency PDs, could be considered in some clinical situations to be more ictal than interictal and may carry a potential risk of neuronal injury, as found in SE.^10^ In this context, PMA may be suggestive of NCSE when EEG findings alone according to the current criteria are inconclusive. These suggestions should be considered with caution, as the numbers of patients in the current study are relatively low and some comparisons were not significantly different after post hoc analysis. Further multicenter validation studies with larger numbers of patients would shed more light on the issue of “ictality” of low‐frequency PD/SW.

### 4.2 Are IIC and NCSE different entities or essentially a spectrum of one entity?

Greater likelihood of hyperperfusion in patients with IIC and low frequency of PD/SW aligns with the concept of gradual “metabolic compensation.”^8^, ^27^, ^28^, ^29^ Increased ictal perfusion in metabolically compensated cases may be reversible. However, in cases of sustained ictal activity it can be persistent, and if energy demand is impaired it can result in diffusion restriction and swelling.^20^ We could speculate that in the case of low‐frequency PD/SW, high metabolic demand reflected in focal cerebral hyperperfusion may serve as an alarming sign of potential later “decompensation,” which may then lead to intracellular diffusion restriction and eventual neuronal damage. In a study conducted on 90 comatose patients with nontraumatic subarachnoid hemorrhage, periodic sharp waves in frequencies above 2 Hz correlated with metabolic decompensation, whereas those of lower frequencies indicated increased cerebral blood flow to compensate seizure‐induced hypoxia.^30^ However, the number of patients with lateralized and generalized PD/SW was small in this highly selected population. In a positron emission tomography (PET) study on patients with lateralized PD/SW, metabolism increased by a median of 100% if patients had PD/SW of 1 Hz, and metabolism went up by a median of 309% for each increase in frequency by 1 Hz compared to the baseline.^31^ Again, only very few patients had lateralized PD/SW at a frequency higher than 2.5 Hz. In a more recent study by Gijs et al., no association of hypermetabolism on PET and frequencies of lateralized PD/SW was found, suggesting that the frequency alone does not reflect the metabolic burden.^32^

### 4.3 Is the 2.5‐Hz boundary applicable to all types of NCSE?

In a study on 4772 patients undergoing continuous EEG, lateralized PD/SW were associated with seizures, irrespective of their frequency, whereas generalized PD/SW correlated with seizures only at frequencies of ≥2 Hz.^17^ Koutroumanidis and Sakellariou^33^ suggested that low‐frequency nonevolving generalized PD/SW could represent ictal patterns, based on treatment response to ASMs, indicating that NCSE may be a relevant diagnosis in patients with cerebral hypoxia.

Interestingly, the Salzburg EEG criteria for electrographic SE set the threshold at epileptiform discharges exceeding 2.5 Hz.^2^ This threshold may underestimate patients with lower frequencies as “possible NCSE” or IIC without considering clinical features such as etiology, with the risk of underdiagnosing true NCSE cases.^8^

It is important to note that the validation study of Salzburg criteria^21^ included patients with prior epilepsy diagnoses (38% of the validation cohort), who typically exhibit high‐frequency PD/SW on EEG. In this context, it is worth acknowledging that our cohort reflects selection bias, as MRI was performed more often in patients with symptomatic etiologies (90%) than in those with a known history of epilepsy (10%). Despite the limited number of patients with acute‐triggering factors in epilepsy as etiology, the LCA identified two distinct classes in our cohort: one predominantly composed of patients with acute‐triggering factors in epilepsy, characterized by nonunilateral EEG patterns and PD/SW frequencies of >2.5 Hz, and another class with low‐frequency PD/SW and diverse etiologies, but predominantly symptomatic, typically displaying unilateral EEG patterns. Additionally, analysis of PMA showed significant difference between the two classes, with PMA occurring more frequently in patients with low‐frequency PD/SW and symptomatic etiologies. This raises the question of whether a frequency of 2.5 Hz is truly an optimal threshold for patients with symptomatic etiologies, or if a reevaluation of this parameter, including etiology as part of the equation, is necessary for better diagnostic accuracy.

We hypothesize that PMAs are not an absolute marker of SE but rather reflect one dimension of “ictality,” namely, the potential for neuronal injury. In this context, etiology may influence functional brain reserve, thereby modulating susceptibility to damage induced by epileptiform discharges.^8^, ^25^ For instance, electrographic seizures may be deleterious by increasing intracranial pressure in patients with traumatic brain injury, whose autoregulatory mechanisms are often impaired.^34^ Conversely, patients with known epilepsy, experiencing SE due to acute‐triggering factors in epilepsy as etiology, tend to exhibit favorable clinical outcomes.^25^ Discrepancies with other studies that link high‐frequency patterns to a greater likelihood of seizures may be attributable to the expected underrepresentation of patients with known epilepsy in intensive care unit cohorts.^17^ The results of our exploratory analysis should be, however, carefully interpreted, as they need an external validation on larger numbers of patients.

### 4.4 Redefining EEG in NCSE toward an etiology‐based categorization

Our findings underscore the potential value of incorporating an etiology‐driven approach to EEG interpretation. Our results suggest that in certain etiologies, EEG patterns with frequencies below the conventional threshold may still be associated with PMA. Accordingly, we propose that EEG interpretation in suspected NCSE could benefit from greater integration of underlying etiology,^35^ which may improve diagnostic accuracy and support more individualized management strategies.

### 4.5 Limitations

This study has several limitations. First, it was conducted at a single center, which may limit the generalizability of the findings. Additionally, the sample size in certain subgroups, particularly those with triggered factors associated with epilepsy, was small, potentially affecting the statistical power. A significant limitation is the potential selection bias, as MRI was more frequently performed in patients without a history of epilepsy, leading to a higher proportion of patients with acute symptomatic etiologies, which limits the generalizability to IIC in patients with known epilepsy. This also limited the subgroup analysis per etiology during LCA. MRI was not performed in 41% of patients, and 24% either had nonprotocol MRI or had MRI done >48 h after the SE/IIC diagnosis. These exclusions introduce serious selection bias; however, as this study was on PMA, we wanted to image patients during the time window that provides the best chance of capturing PMA. Furthermore, the dynamic nature of EEG patterns, which can evolve over time, presents another limitation. Absence of continuous EEG immediately before and after the MRI limited the evaluation of dynamic EEG changes during the peri‐MRI period.

We observed a moderate agreement on EEG patterns between independent experts from different centers. This is, however, in line with observations related to some EEG patterns that could be equivocal.^36^, ^37^ Finally, although the difference in median time lapse between EEG and MRI was not statistically significant, the IQRs were as wide as 24 h. As the patients received treatment for either SE or IIC between EEG and MRI, the temporal relationship between these two test findings should be cautiously interpreted.

Given the exploratory nature of the analyses, the observed differences should be considered preliminary and warrant confirmation in independent datasets. It also should be noticed that one of the classes identified by the LCA contains only 8.3% of the observed sample, which does not make the model ideal for such an analysis.

## 5 CONCLUSIONS

Our findings suggest an overlap in neuroimaging profiles of patients with SE and IIC, challenging the strict separation of these entities. Increased rates of hyperperfusion in association with low‐frequency epileptiform discharges serve as an indicator for increased metabolic demand, which may potentially decompensate and lead to a neuronal injury. Additionally, our results suggest considering etiology while interpreting EEG in patients with IIC.

## AUTHOR CONTRIBUTIONS

Pilar Bosque Varela, Lukas Machegger, Wanda Lauth, and Giorgi Kuchukhidze contributed significantly to conception and design of the presented paper, acquisition, analysis, and interpretation of the data, and drafting of the paper. Panagiota Eleni Tsalouchidou, Susanne Knake, Georg Zimmermann, Nicolas Jannone‐Pedro, Giada Giovannini, Stefano Meletti, Adrian Ridski Harsono, Fabio Rossini, Markus Leitinger, Johannes Pfaff, and Sándor Beniczky contributed to analysis of data and revising the paper for intellectual content. Giorgi Kuchukhidze and Eugen Trinka contributed significantly to conception of the study and interpretation of the results and gave final approval of the submitted version of the manuscript.

## CONFLICT OF INTEREST STATEMENT

E.T. reports personal fees from EVER Pharma, Marinus, Arvelle, Angelini, Argenx, Alexion, Medtronic, BIAL–Portela & Cª, NewBridge, GL Pharma, GlaxoSmithKline, Boehringer Ingelheim, LivaNova, Eisai, UCB, Biogen, Rapport Sanofi, Jazz Pharmaceuticals, Stoke Therapeutics, and Actavis. He is codirector of the European Consortium on Epilepsy Trials. His institution has received grants from Biogen, UCB Pharma, Eisai, Red Bull, Merck, Bayer, the European Union, Austrian Research Fund (FWF) Osterreichischer Fond zur Wissenschaftsforderung, Bundesministerium für Wissenschaft und Forschung, and Jubiläumsfond der Österreichischen Nationalbank (none related to the present work). G.K. has received research grants from the FWF (project number KLI 969) and Paracelsus Medical University (project number 2021‐UP‐003‐Kuchukhidze); and travel grants and honoraria from UCB, Jazz Pharmaceuticals, Angelini, and Novartis. P.B.V. has received travel grants and honoraria from UCB. N.J.‐P. has received travel grants and honoraria from UCB, not related to the present work. P.E.T. has received research grants from the German Society for Epileptology (Otfrid‐Foerster Stipendium, DGfE), as well as travel grants from UCB and Angelini, and honoraria for lectures from UCB, none of which is related to the present work. S.M. has received research grant support from the Ministry of Health; and has received personal compensation as a scientific advisory board member from UCB, Jazz pharmaceuticals, Angelini, and Eisai. S.K. has received speaker's honoraria from Angelini, Bial, Eisai, Desitin, Merck Serono, and UCB, not related to the present work. None of the other authors has any conflict of interest to disclose. We confirm that we have read the Journal's position on issues involved in ethical publication and affirm that this report is consistent with those guidelines.

## Supporting information


Data S1.

## Data Availability

The corresponding author takes full responsibility for the data, the analyses and interpretation, and the conduct of the research; the corresponding author has full access to all of the data and has the right to publish any and all data separate and apart from any sponsor.
