# Supplementary material for: Ictal–interictal continuum and status epilepticus: Two sides of the same coin? A prospective magnetic resonance imaging study
Source: Epilepsia. 2026 Feb 2;67(5):2377–88. doi: 10.1002/epi.70131 (PMC13179647; doi:10.1002/epi.70131)
Supplement: Supplementary file 1 — Data S1. [file EPI-67-2377-s001.docx]

**Supplementary material:**

**MRI protocol for patients with status epilepticus**

**DWI**: spin echo-EPI diffusion imaging was used to acquire 28 slices with echo time (TE) 47 ms, repetition time (TR) 3.051 ms, field of view (FOV) 230 × 230 mm, and voxel 2.05 × 2.56 mm, with a slice thickness of 4 mm and a gap between the slices of 1 mm. The diffusion sequence was acquired with four b-values of 0, 333, 666, and 1.000 s/mm² to calculate the conventional ADC map. Diffusion gradients were applied in three directions.

**FLAIR**: MS TSE was used to acquire 28 slices with echo time (TE) 125 ms, repetition time (TR) 10000 ms, inversion time 2800 ms (TI), field of view (FOV) 560 mm × 560 mm, and voxel 0.65 × 1.13 mm, with a slice thickness of 4 mm and a gap between the slices of 5 mm.

**T1 3D Sequence**: T1W_FFE was obtained in a sagittal alignment with echo time (TE) 4.04 ms, repetition time (TR) 8.66 ms, field of view (FOV) 320 mm × 320 mm, and voxel 1 × 1 × 1 mm. T1W_FFE was done before and after administration of gadolinium.

**pCASL**: MS FFE single shot EPI with 39 as EPI factor. 20 slices, field of view (FOV) 240 mm x 240 mm x 119 mm, voxel 2.75 mm x 2.75 mm, with a slice thickness of 5 mm and a gap of 1 mm. Scan duration 4:28 minutes, fat saturation SPIR, temporal resolution 30 pictures, labeling distance 90, post labeling delay 1800 ms which was adapted to the age of patients. TR 4324 ms, TE 13 ms. This scan was operating in 1st level controlled mode.

**T2* perfusion**: Epi FFE with 40 dynamic scans had a voxel size of 2.33 x 2.33 x 4.00mm and a scan duration of 1:14. Dynamic scan time was “shortest”. TR/TE was 1714/40 ms with SPIR fat suppression. Gadolinium application was started manually.

**TOF**: 3D FFE TOF was done in 200 slices. Scan duration 4:08. Field of view 200 x 200 x 140 mm, voxel size 0.54 x 0.82 x 1.40 mm and chunk thickness of 14 mm. TR/TE was 23/3.5 ms. One parallel suppression pulse in "head" position with a thickness of 30 mm a gap of 10 mm was used.

**DTI**: single shot EPI diffusion with echo time (TE) 74.43 ms, repetition time (TR) 6398.99 ms, field of view (FOV) 128 × 128 mm, and voxel 2.00 × 2.04 mm, with a slice thickness of 2 mm and a gap between the slices of 0 mm. The diffusion sequence was acquired with two b-values of 0 and 800 s/mm²; 16 spatial directions were measured.

**Supplementary Table 1: PMA spectrum SE-PM to IIC vs. SE-PM to NCSE**

| **PMA spectrum** | **SE-PM to IIC**  **N=15** | **SE-PM to NCSE**  **N=10** | **OR, 95% CI (p value)** |
| --- | --- | --- | --- |
| **Peri-ictal hyperperfusion** | 10 (67%) | 7 (70%) | 0.86, 0.09-6.35 (1) |
| **Diffusion restriction** | 8 (53%) | 5 (50%) | 1.13, 0.17-7.49 (1) |
| **FLAIR-hyperintensity** | 8 (53%) | 5 (50%) | 1.13, 0.17-7.49 (1) |

**Abbreviations:** PMA: Peri-ictal MRI abnormalities; SE-PM: Status epilepticus with prominent motor symptoms; IIC: Ictal-interictal continuum; NCSE: Non-convulsive status epilepticus; OR: Odds ratio; CI: Confidence interval; FLAIR: Fluid attenuated inversion recovery

**Supplementary Table 2: PMA spectrum among clusters of patients based on latent cluster analysis**

| **PMA spectrum** | **Class 1, N=11** | **Class 2, N=122** | **OR, 95% CI** | **p value** |
| --- | --- | --- | --- | --- |
| **Peri-ictal hyperperfusion** | 2 (18%) | 61 (50%) | 4.46 (0.87-44.05) | 0.05 |
| **Diffusion restriction** | 1 (9%) | 49 (40%) | 6.64 (0.89-296) | 0.05 |
| **FLAIR-hyperintensity** | 1 (9%) | 44 (36%) | 5.59 (0.75-250) | 0.09 |

**Abbreviations:** PMA: Peri-ictal MRI abnormalities; OR: Odds ratio; CI: Confidence interval; FLAIR: Fluid attenuated inversion recovery
